# Supplementary material for: Role and mechanism of NCAPD3 in promoting malignant behaviors in gastric cancer
Source: Front Pharmacol. 2024 Apr 22;15:1341039. doi: 10.3389/fphar.2024.1341039 (PMC11070777; doi:10.3389/fphar.2024.1341039)
Supplement: Supplementary file 11 [file DataSheet2.ZIP › GSEA/Oncogenic signature/my_analysis.Gsea.1599461962066/MEL18_DN.V1_UP.html]

Details for gene set MEL18\_DN.V1\_UP[GSEA]

|  || Dataset | filtered\_dataset.sample\_info.cls#WT\_versus\_NCAPD3\_MUT |
| Phenotype | sample\_info.cls#WT\_versus\_NCAPD3\_MUT |
| Upregulated in class | NCAPD3\_MUT |
| GeneSet | MEL18\_DN.V1\_UP |
| Enrichment Score (ES) | -0.42256725 |
| Normalized Enrichment Score (NES) | -2.0413263 |
| Nominal p-value | 0.0049261083 |
| FDR q-value | 0.020260772 |
| FWER p-Value | 0.1 |
Table: GSEA Results Summary

  

Fig 1: Enrichment plot: MEL18\_DN.V1\_UP      
 Profile of the Running ES Score & Positions of GeneSet Members on the Rank Ordered List

  

| SYMBOL | TITLE | RANK IN GENE LIST | RANK METRIC SCORE | RUNNING ES | CORE ENRICHMENT || 1 | 55240 | STEAP3 | 169 | 0.710 | -0.0650 | No |
| 2 | 6573 | SLC19A1 | 231 | 0.650 | -0.0570 | No |
| 3 | 4297 | KMT2A | 337 | 0.573 | -0.0868 | No |
| 4 | 2919 | CXCL1 | 405 | 0.534 | -0.0923 | No |
| 5 | 64866 | CDCP1 | 515 | 0.466 | -0.1336 | No |
| 6 | 8140 | SLC7A5 | 890 | -0.305 | -0.3786 | No |
| 7 | 5329 | PLAUR | 952 | -0.373 | -0.3928 | Yes |
| 8 | 10123 | ARL4C | 957 | -0.377 | -0.3655 | Yes |
| 9 | 3914 | LAMB3 | 1015 | -0.414 | -0.3735 | Yes |
| 10 | 9308 | CD83 | 1025 | -0.423 | -0.3461 | Yes |
| 11 | 23046 | KIF21B | 1034 | -0.433 | -0.3173 | Yes |
| 12 | 51200 | CPA4 | 1035 | -0.434 | -0.2826 | Yes |
| 13 | 9076 | CLDN1 | 1084 | -0.463 | -0.2801 | Yes |
| 14 | 5834 | PYGB | 1096 | -0.475 | -0.2500 | Yes |
| 15 | 54541 | DDIT4 | 1133 | -0.502 | -0.2358 | Yes |
| 16 | 2152 | F3 | 1188 | -0.559 | -0.2300 | Yes |
| 17 | 23657 | SLC7A11 | 1205 | -0.581 | -0.1951 | Yes |
| 18 | 8061 | FOSL1 | 1222 | -0.597 | -0.1588 | Yes |
| 19 | 1893 | ECM1 | 1250 | -0.637 | -0.1274 | Yes |
| 20 | 8744 | TNFSF9 | 1305 | -0.708 | -0.1097 | Yes |
| 21 | 1839 | HBEGF | 1328 | -0.751 | -0.0655 | Yes |
| 22 | 6509 | SLC1A4 | 1330 | -0.752 | -0.0061 | Yes |
| 23 | 7422 | VEGFA | 1346 | -0.788 | 0.0461 | Yes |
Table: GSEA details [plain text format]

  

Fig 2: MEL18\_DN.V1\_UP      
 Blue-Pink O' Gram in the Space of the Analyzed GeneSet

  

Fig 3: MEL18\_DN.V1\_UP: Random ES distribution      
 Gene set null distribution of ES for **MEL18\_DN.V1\_UP**
